# Detection of *Strongylus vulgaris* in equine faecal samples by real-time PCR and larval culture – method comparison and occurrence assessment

**DOI:** 10.1186/s12917-016-0918-y

**Published:** 2017-01-11

**Authors:** A. Kaspar, K. Pfister, M. K. Nielsen, C. Silaghi, H. Fink, M. C. Scheuerle

**Affiliations:** 1 Comparative Tropical Medicine and Parasitology, Veterinary Faculty, Ludwig-Maximilians-University, Munich, Germany; 2 Present address: Parasite Consulting GmbH, Wendschatzstrasse 8, CH-3006 Berne, Switzerland; 3 Department of Veterinary Science, M.H. Gluck Equine Research Center, University of Kentucky, Lexington, KY USA; 4 Present address: National Centre of Vector Entomology, Institute for Parasitology, Vetsuisse Faculty, University of Zurich, CH-8006 Zurich, Switzerland; 5 Department of Statistics, Ludwig-Maximilians-University, Munich, Germany; 6 Present address: ParaDocs Laboratory, Ismaning, Germany

**Keywords:** *Strongylus vulgaris*, Strongyle, Equine, Larval culture, Real-time PCR, Germany

## Abstract

**Background:**

*Strongylus vulgaris* has become a rare parasite in Germany during the past 50 years due to the practice of frequent prophylactic anthelmintic therapy. To date, the emerging development of resistance in Cyathostominae and *Parascaris* spp. to numerous equine anthelmintics has changed deworming management and the frequency of anthelmintic usage. In this regard, reliable detection of parasitic infections, especially of the highly pathogenic *S. vulgaris* is essential. In the current study, two diagnostic methods for the detection of infections with *S. vulgaris* were compared and information on the occurrence of this parasite in German horses was gained. For this purpose, faecal samples of 501 horses were screened for *S. vulgaris* with real-time PCR and an additional larval culture was performed in samples of 278 horses. A subset of 26 horses underwent multiple follow-up examinations with both methods in order to evaluate both the persistence of *S. vulgaris* infections and the reproducibility of each diagnostic method.

**Results:**

The real-time PCR revealed *S. vulgaris-*DNA in ten of 501 investigated equine samples (1.9%). The larval culture demonstrated larvae of *S. vulgaris* in three of the 278 samples (1.1%). A direct comparison of the two methods was possible in 321 samples including 43 follow-up examinations with the result of 11 *S. vulgaris*-positive samples by real-time PCR and 4 *S. vulgaris*-positive samples by larval culture. The McNemar’s test (*p*-value = 0.016) revealed a significant difference and the kappa values (0.525) showed a moderate agreement between real-time PCR and larval culture.

**Conclusions:**

The real-time PCR detected a significantly higher proportion of positives of *S. vulgaris* compared to larval culture and should thus be considered as a routine diagnostic method for the detection of *S. vulgaris* in equine samples.

## Background

The increasing resistance of small strongyles (Cyathostominae), *Parascaris* spp*.* (Ascarididae) and *Oxyuris equi* (Oxyuridae) against anthelmintic drugs [1–5] requires major changes in equine parasite control. During the past 50 years, horses were mainly treated prophylactically multiple times per year [6–11]. This approach was based on recommendations, given mainly in order to avoid infections with *Strongylus vulgaris* [12]. Due to the emerging resistance of Cyathostominae to available anthelmintic drugs, an alternative approach based on selective anthelmintic therapy (SAT) has been implemented in some horse farms [13, 14]. The principle of SAT comprises a major change in the frequency of applying anthelmintics with an anti-strongyle spectrum based on the treatment of horses with a faecal egg count (FEC) of strongyle eggs above a certain threshold, determined with a quantitative faecal egg count method, such as the McMaster [3, 15–18]. Nevertheless, for reliable detection of infections with other important equine parasite species like *Anoplocephala perfoliata*, *O. equi,* and *Strongylus* spp. (large strongyles, Strongylinae) further specific diagnostic tests should be applied [13]*.*

The intensive anthelmintic treatment regime of the last decades might be responsible for the current low occurrence of *S. vulgaris* (0.2–1.3%) recently determined by larval culture in German horses [10, 19–21]. However, a reliable diagnostic method for the differentiation of *S. vulgaris* from Cyathostominae is essential because of the significant pathology caused by the migration of *S. vulgaris*-larvae in the mesenteric arteries. Associated pathogenic effects like intestinal infarction, peritonitis, verminous arteritis, thrombosis and embolism can provoke symptoms such as colic, hind-leg lameness, neurological abnormalities and lesions in the heart, liver or kidney which might be lethal in the worst case [22–24]*.*

Currently, the standard method for the differentiation of Cyathostominae and Strongylinae is the determination of morphological differences of third-stage larvae (L3) after larval culture since their egg morphology is similar and thus impossible to distinguish [25, 26]. However, even though this method has the advantage of being cost-efficient, it is also very time-consuming due to the developmental period of the strongyle egg to the L3 and the subsequent morphological differentiation which should be performed by experienced qualified personnel [27–29].

To date, detection of *S. vulgaris* is possible with conventional and real-time PCRs in faecal samples as well as a serum Enzyme linked Immunosorbent Assay (ELISA) [30–32].

According to previously conducted studies, both the conventional and the real-time PCR provide high specificity and sensitivity [30, 33–37]. Moreover, Nielsen et al. [30] claimed the real-time PCR as a potential standard method for a reliable detection of *S. vulgaris* [30]. Therefore, the objectives of the present study were to evaluate the detection of *S. vulgaris* via real-time PCR in comparison to the standard diagnostic method (larval culture) and to add information about the occurrence of *S. vulgaris* in German horses detected by real-time PCR and larval culture.

## Methods

### Faecal samples

In total, 1455 equine faecal samples from 91 German farms were collected from March 2013 to May 2014. All samples were obtained from the Diagnostic Centre of the Chair of Comparative Tropical Medicine and Parasitology, LMU Munich, Germany. Every sample was analysed using a modified McMaster method with a detection limit of 20 eggs per gram faeces (EPG) and the combined-sedimentation-flotation method [17]. Samples with a minimum of 20 EPG were selected.

**Fig. 1:**
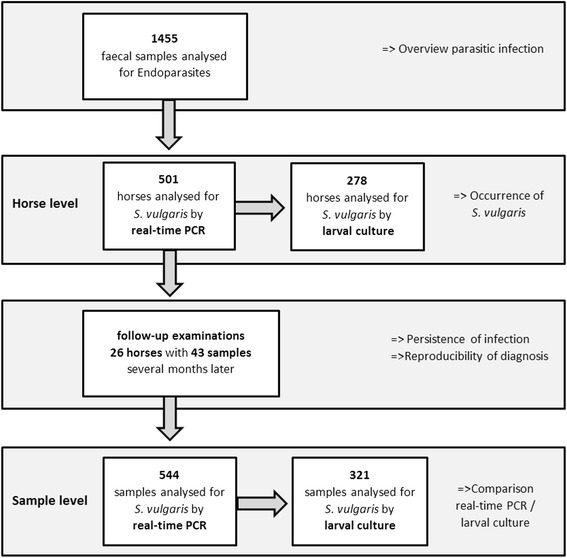
Overview of the division of equine faecal samples and the corresponding motivation

Assuming a German horse population of 1.1 million [38] with a prevalence for an infection with *S. vulgaris* of 1% [3, 10, 19–21], a minimum of 459 horses had to be investigated since in this case the probability to detect at least one infected horse was above 99% [39]. Thus, samples of 501 of 1455 horses (horse level, 34.4%) were investigated by real-time PCR for an infection with *S. vulgaris* (Fig. 1) in the present study.

For 278 of the 501 horses, a sufficient amount of faecal material was available for the preparation of a larval culture in order to differentiate the larvae.

In 26 of the 501 investigated horses, follow-up examinations were carried out 4–6 months after the initial examination. In this context, a total of 43 samples of the 26 horses from six different farms were analysed by real-time PCR and larval culture. Follow-up examinations were performed if either the real-time PCR-result was positive for *S. vulgaris* (Table 1) or non-determinable larvae were present in the first larval culture examination. In the case of two horses which were *S. vulgaris* positive in the first examination, the whole horse group was tested also for *S. vulgaris*. With these examinations, the dissemination of *S. vulgaris* should be verified at horses living on the same holding.

**Table 1:** Dates of initial and follow-up examinations of four real-time PCR-positive horses

Horse	1	2	3	4
Date of first examination	2013/05/27	2013/06/04	2013/08/06	2013/06/13
Date of follow-up examinations	2013/09/16	2013/10/30	2014/02/11	2014/02/07
		2013/10/31	2014/02/12	2014/02/08
		2013/11/01	2014/02/13	
		2013/11/02	2014/02/14	
		2013/11/03	2014/02/15	
		2013/11/04	2014/02/16	
		2013/11/05	2014/02/17	
			2014/02/18	
			2014/02/19	
			2014/02/20	
			2014/02/21	

None of the horses that underwent a follow-up examination had been dewormed until the date of the retest. Thus, a total of 544 samples were analysed by real-time PCR and a total of 321 samples were analysed by larval culture (sample level, Fig. 1).

The total data pool was separated into two data sets for the different analyses either on sample or on horse level (Fig. 1). Furthermore, prevalence analyses were performed on horse and on farm level. As soon as one horse was tested *S. vulgaris*-positive, its farm of origin was classified as positive on the farm level.

As far as possible, information was collected on every investigated horse, namely age, breed, gender and the date of the last anthelmintic treatment.

### Egg detection, concentration and isolation

Gastrointestinal strongyle eggs were isolated from faecal samples using a combined-sedimentation-flotation technique [17]. At first, 40 g of faeces were mixed with 500 ml of water, then filtered through a 300 μm sieve and sedimented at 10 °C for 12 h. The sediment was divided and transferred into two centrifuge tubes – one with saturated sugar solution (specific weight/solution density 1.27 +/− 0.01) and one with saturated zinc sulphate solution (ZnSO_4_, specific weight/solution density 1.28 +/− 0.01; Carl Roth GmbH and Co. KG, Karlsruhe, Germany) and centrifuged for 5 min at 635 g.

In case of a positive microscopic diagnosis of strongyle eggs, the egg counts were classified as sporadic (0–300 eggs), numerous (301–3000) or plentiful (>3000).

The eggs were rinsed from the cover slip with distilled water, poured into a beaker and into a centrifuge tube (conical shape) which was then centrifuged at 635 g for 10 min. The remaining liquid was withdrawn by suction. The remaining sediment was transferred into a 1.5 ml Eppendorf tube and then centrifuged one more time at 10,621 g for 3 min. The remaining liquid was withdrawn by suction and the sediment was frozen in a dry state at −20 °C.

### Larval identification

Larval cultures modified after Roberts and O’Sullivan [40] were carried out for the morphological determination of *S. vulgaris* larvae. Ten grams of faeces were incubated at room temperature for 14 days. During these 14 days, the samples were ventilated every day for 1 h and moistened to avoid desiccation of the faeces. The Baermann-Wetzel technique was used to isolate the L3 [17].

After the migration time of 24 h at room temperature, 10 ml of the liquid was drawn off from the Baermann funnel. For concentration of the larvae, the liquid was stored in a centrifuge tube at 10 °C for another 24 h. Subsequently, the supernatant was withdrawn by suction and 2 ml were left in the tube. All larvae contained in the sediment were counted (L/10 g = larvae per 10 g faeces) and morphologically determined via microscope. For immobilisation of the larvae, one drop of Lugol’s iodine was added to one drop of the larval suspension and investigated immediately. The morphological criteria were determined according to Bürger et al. [41] and Boch et al. [42].

### DNA extraction from isolated eggs

The extraction was carried out according to the manufacturer’s instruction (QIAamp® DNA Mini and Blood Mini Handbook) according to the blood or body fluid spin protocol (Qiagen, Hilden, Germany). Briefly, before extraction the sediment containing the strongyle eggs was incubated in an ultrasonic bath with 150 μl PBS (phosphate buffered saline) for 4 min followed by a freezing step for 20 min at −20 °C and a repetition of the incubation step. The DNA was eluted with 200 μl elution buffer. DNA concentration (ng/μl) and quality of each sample were analysed with the Nano-Drop ND-1000 Spectrophotometer (PeqLab, Erlangen, Germany). The samples were stored at –20 °C until used for PCR analysis.

### Molecular analyses

#### Real-time PCR

Samples were screened for *S. vulgaris* by real-time PCR using the AB-7500 Real Time PCR System (Applied Biosystems, Darmstadt, Germany). PCR targeted rDNA sequences of the second internal transcribed spacer (ITS-2: 169 bp) of Cyathostominae and *Strongylus* spp*.* nematodes. The forward primer Sv-f (5′-GTATACATTAAATAGTGTCCCCCATTCTAG-3′), the reverse primer Sv-r (5′-GCAAATATCATTAGATTTGATTCTTCCG-3′) and the modified probe Sv-p 5′-FAM-TGGATTTATTCTCACTACTTAATTGTTTCGCGAC-BHQ1-3′ were used as previously described [30]. The 25 μl reaction volume consisted of 5 μl of template DNA, 2.25 μl (0.9 μM) of each primer (10 μM), 0.5 μl of the probe (0.2 μM) and 15 μl TaqMan Gene Expression Master Mix (Life technologies GmbH, Darmstadt, Germany). A negative and a positive control were added to each PCR reaction. The positive control (DNA extracted from adult worms which were morphologically identified as *S. vulgaris*) was provided by the M. H. Gluck Equine Research Center, University of Kentucky, USA. The following PCR protocol was used: an initial activation at 95 °C for 10 min followed by a set of 40 cycles, each consisting of 15 s at 95 °C and 60 s at 60 °C (AB Systems Standard protocol, Applied Biosystems, Darmstadt, Germany).

To verify the efficiency and the relative sensitivity of the real-time PCR, a standard curve was created according to the AB Systems manufacturer’s instruction (Applied Biosystems, Darmstadt, Germany) with ten fold dilution steps of the positive control (threefold preparation). An analysis was performed up to a dilution step of 1:100,000.

Results were recorded as the mean PCR cycle number at which the fluorescence detection threshold had been exceeded (C_t_). The threshold line was set at the optimal point in the linear phase of the amplification plot. The observation of an exponential rise up to a C_t_ of 37.5 was counted as positive according to the results of the standard curve.

#### Conventional PCR and Sequencing

In order to verify the results of the real-time PCR, an additional conventional PCR with subsequent sequencing of the amplification products was performed with real-time PCR-positive samples using the same primers (100 μM) (Eppendorf Mastercycler MWG Biotech, Ebersberg, Germany).

The reaction mix contained 5 μl of template DNA, 5 μl (1x) of buffer 10x, 1 μl (200 μM) of dNTPS, 0.5 μl (1 μM) of each primer (100 μM), 0.25 μl (1.25 U) of HotStar Taq DNA Polymerase Kit (Qiagen, Hilden, Germany) and water-ultra pure grade filled up to a total volume of 50 μl. The following protocol was used: 5 min at 95 °C for one cycle, 95 °C for 30 s, 49 °C for 30 s and 72 °C for 40 s for 45 cycles, followed by 72 °C for 5 min.

PCR products of the conventional PCR were analysed in 2% agarose gel stained with Gel Red™ nucleic acid stain, 10.000 x in water (both from Biotium, Hayward, USA). The DNA Gene ruler 100 bp Plus DNA ladder was used for sizing and quantification of the PCR products. The visualisation of gel images was performed with a gel documentation system (Peqlab, Erlangen, Germany).

The positive PCR products were purified using the QIAquick PCR Purification Kit (Qiagen, Hilden, Germany) following the manufacturer’s protocol. Forward and reverse sequencing was performed by Eurofins MWG Operon (Ebersberg, Germany). Reverse sequences were reversed, complemented, and aligned to the forward sequences using online tools (Reverse Complement: http://www.bioinformatics.org/sms/rev_comp.html, Clustal Omega: https://www.ebi.ac.uk/Tools/msa/clustalo). Database searches and sequence comparisons were done with BLAST provided by the National Center for Biotechnology Information (BLAST: http://blast.ncbi.nlm.nih.gov/Blast.cgi).

## Statistical Analyses

Statistical analyses were performed using IBM® SPSS® software version 22.0 (IBM Corporation, Armonk, USA) and Microsoft Excel 2010 (Microsoft Corporation, Seattle, USA). Since our data did not satisfy normality assumptions, classical non-parametric methods were used. The correlation between EPG and L/10 g faeces was calculated using a Spearman’s rank correlation coefficient. In order to determine the statistical difference of the capability of the real-time PCR and the larval culture to detect a *S. vulgaris* positive sample, a McNemar test was performed for comparison of paired proportions and Kappa values were calculated for an evaluation of agreement between the two tests. All statistical analyses were interpreted as statistically significant up to the *p*-value < 0.05.

## Results

### Sample origin

Out of the 1455 samples, strongyle faecal egg counts (FECs) of 804 samples (55.3%) were positive ranging from <20 to 11,080 EPG. An EPG of >200 was recorded in 348 samples (23.9%).

Of the 804 faecal egg count positive samples, 544 samples (20–11,080 EPG; mean: 482 EPG; median: 200 EPG) from 501 horses were randomly selected for further investigation with real-time PCR. The number of investigated horses originating from 91 farms ranged from 1 to 112 per farm.

The FEC of the real-time PCR *S. vulgaris-*positive samples, ranged from 20 to 1720 EPG. The FEC of one sample could not be determined due to the low amount of faecal material available. However, it was included in the study due to the numerous strongyle eggs detected in the combined-sedimentation-flotation.

Of those 501 animals, the age was known for 374 and the gender was known for 425 horses.

The age of the horses ranged from 1 to 35 years with an average age of 12 years. Samples originating from 174 mares (40.9%), 246 geldings (57.9%) and five stallions (1.2%) were examined. On average for 398 horses, the last anthelmintic treatment had been performed 11 months (range 2–196 months) prior to enrolment into the present study.

### Larval culture

Larval culture were performed and evaluated from 321 faecal samples originating from 278 horses (including follow-up examinations). A total of 451,832 Cyathostominae larvae (L3) were detected in 93.5% of the cultures (100.0% of the farm level). Between 1 and 31,251 larvae per 10 g faeces were counted. The EPG and the L/10 g faeces correlated with a Spearman’s rank correlation of 0.826 (*p* = 0.000).

A total of 11 *S. vulgaris* larvae were detected in altogether four samples (sample level).

Regarding the horse level, *S. vulgaris* was found in 3 of 278 horses (1.1%). *S. vulgaris*-positive horses originated from 3 out of 62 investigated farms (4.8%).

The occurrence of different larval stages in a total of 321 larval cultures was documented in detail. Strongylinae larvae in first- (L1) and second-stage (L2) were found in 52 samples in a range of sporadic (max. 4 larvae/sample) and in four samples in a range of numerous (>15 larvae). Additionally, empty sheaths of Strongylinae larvae were found in five of the 321 samples (max. 3/sample). Larvae of *Triodontophorus* sp. were found in nine samples, larvae of *Trichostrongylus axei* in three samples and larvae of free-living nematodes were present in 15 samples.

### Real-time PCR

The real-time PCR was positive in 13 of 544 investigated samples (2.4%; Table 2).

**Table 2:** Summarized results of real-time PCR-positive samples (FEC, combined-sedimentation-flotation, larval culture** and C_t_-value)

Sample-No.	FEC	Combined-sedimentation-flotation*	Larval culture Cyathostominae**	Larval culture *S. vulgaris* **	C_t_
21	20	+	16	3	27.58
144^a^	20	+	5	4	24.94
235	1040	+++	---	---	33.49
318	100	+	533	0	26.90
334	900	+++	1256	0	35.81
355	---	++	---	---	27.67
375	40	+	28	0	35.99
412	1720	+++	103	0	23.40
448^b^	20	+	34	0	36.95
451^c^	20	+	57	0	35.07
460	200	++	535	0	35.75
497	480	+++	1111	3	29.87
520	680	+++	3028	1	34.97

FEC = faecal egg count; Ct = cycle threshold

* + = sporadic eggs; ++ = numerous; +++ = plentiful

** Number of larvae of Cyathostominae or *S. vulgaris* /10 g faeces

--- diagnostic method not performed

^a^follow-up examination of sample-No. 21, 2013/09/16

^b^sample-No. 375, 2014/02/12 and

^c^sample-No: 375, 2014/02/15

With regard to the horse level of 501 horses, the occurrence of *S. vulgaris* obtained by real-time PCR was 1.9% (10/501). *S. vulgaris-*positive horses originated from 10 of 91 investigated farms (10.9%). On each farm only one horse was tested positive for *S. vulgaris.*

The standard curve revealed an efficiency of 90.5% (slope -3.571; R^2^ 0.996). A positive analysis was performed with a dilution of up to 1:10,000 (y-Inter 37.428) of the positive control in order to estimate the relative sensitivity. Due to an unknown amount of DNA in the positive control, no reference value was available and it was not possible to analyse a quantitative potential of the real-time PCR. There was no statistically significant correlation between the number of *S. vulgaris* larvae within one sample and the corresponding C_t_-value since the total amount of samples was too small. One PCR-positive sample containing a single larva of *S. vulgaris* had a C_t_-value of 34.97, which is higher compared to the C_t_-values of 24.94 to 29.87 obtained by the other three PCR-positive samples containing three or four larvae in the culture.

### Follow-up examinations

Four initially *S. vulgaris-*positive horses were retested in follow-up examinations. The follow-up samples of two horses tested positive on a daily basis. Horse 1 (Table 1) was tested positive in the single follow-up examination (Table 2) which was performed 4 months after the initial examination. The follow-up examination of horse 3 (Table 1) was carried out 6 months after the initial examination. In this context faecal samples were taken and analysed for *S. vulgaris* on 11 consecutive days. In addition to the initial investigation, *S. vulgaris* was also detected in the faecal samples collected on day 1 and 4 (Table 2). The other follow-up examinations of all horses that were retested were negative for *S. vulgaris.* However, the low number of follow-up examined horses in this study did not allow for a statistical analysis.

### Comparison of real-time PCR and larval culture

A direct comparison between real-time PCR and larval culture could be drawn for 321 samples of the sample level. The results of the latter two methods differed significantly according to the McNemar’s test (0.016) (Table 3). This is in line with Cohen’s kappa which shows a value of 0.525 indicating only moderate agreement.

**Table 3:** Comparison of results of larval culture and real-time PCR

	PCR –	PCR +	Total
Larval culture –	310	7	317
Larval culture +	0	4	4
Total	310	11	321

κ-value: 0.525; 95% confidence interval: [0.219–0.831]; McNemar’s test: *p*-value = 0.016

### Conventional PCR and Sequencing

Interpretable sequencing results were obtained from all 13 isolates. Two of them were 100% homologous with a sequence of *S. vulgaris* from a horse from Australia (GenBank accession no. X77863.1) [33]. Eleven sequences showed a 99% similarity to the latter sequences containing a single nucleotide substitution (C instead of G at bp 61).

Sequences determined at the ITS-2 region in this work were deposited in GenBank under the following accession numbers: KT250609 – KT250621.

## Discussion

*S. vulgaris* was detected in three of 278 investigated horses (1.1%) by larval culture and in 10 of 501 investigated horses (1.9%) by real-time PCR. This low occurrence of *S. vulgaris* is in line with previously conducted studies investigating faecal samples from German horses obtained by larval culture with a prevalence ranging from 0.2 to 1.3% [10, 19–21]. A comparable prevalence has also been reported in Switzerland [3].

In contrast, studies from other countries revealed a higher prevalence for *S. vulgaris* both by larval culture and by PCR. In a study from Denmark, Nielsen et al. [36] screened 663 horses from 42 farms via larval culture and real-time PCR with a result of 113 *S. vulgaris*-positive horses (17.5%). In an investigation of horses from Poland by necropsy, nematodes were isolated from the intestine and differentiated revealing a prevalence of 22.8% (165/725) for *S. vulgaris* [43]. An even higher prevalence of 41.3% (19/46) was detected in horses from Sardinia, Italy, via larval culture [44]. In comparison with data from Germany, Bracken et al. [45] found a higher prevalence of infection with *S. vulgaris* in Danish horses not only on the horse level with 13.6% (45/331) but also on the farm level with 72.2% (13/18). A comparable result on the farm level was reported by Nielsen et al. with a prevalence of 64.3% (27/42) determined by larval culture [18].

In the present study, *S. vulgaris* was detected in ten out of 91 investigated farms (10.9%). A low occurrence of *S. vulgaris* in farms from Germany was further demonstrated by the fact that both the real time PCR and the larval culture revealed a single *S. vulgaris*-positive horse per farm, solely. These findings are in agreement with another recent German prevalence survey, which reported a farm level of 1.04% (2/192) and detected only a single *S. vulgaris* positive horse per farm using larval culture [21].

The intensive anthelmintic treatment regime of the last decades as well as the long prepatent period of six to 7 months might be responsible for the current low occurrence of *S. vulgaris* in Germany [3, 46, 47]. Based on these arguments, a low occurrence for *S. vulgaris* under 5% was reported by Hertzberg et al. [3] for horses living in Switzerland. Despite the intensive anthelmintic treatment regime of the last decades, the occurrence of *S. vulgaris* still persists on a low level. A possible reason for this persistence could be a non-complete larvicidal efficacy of ivermectin, as suggested by Nielsen et al. due to his study results in 2014 [48].

Different surveys have pointed out that the number of Cyathostominae larvae in larval cultures is higher compared to the number of *S. vulgaris* larvae [21, 23, 29, 49]. For example, Ogbourne and Duncan [23] refer to a wide variety of Strongyle species in equine faecal samples comprising larvae of *S. vulgaris* in less than 10%. Bellaw and Nielsen [29] reported that approximately 1.0% of detected larvae were L3 of *S. vulgaris* (1486 *S. vulgaris* vs. 142,725 Cyathostominae larvae). In the present study, 0.16% of all larvae in 13 *S. vulgaris*-positive samples were L3 of *S. vulgaris* (11 *S. vulgaris* larvae vs. 6706 Cyathostominae larvae). Both the marginal number of counted *S. vulgaris*-larvae in the larval culture and the finding of only one *S. vulgaris*-positive horse per farm might be explained by a low infection rate and by a low shedding of *S. vulgaris* eggs of infected horses.

Accordingly, false negative results might occur in samples comprising a low number of *S.vulgaris* eggs. Thus, the dependence on the presence of eggs in the investigated faecal sample is the major disadvantage of the diagnosis of *S. vulgaris* with coprological methods like real-time PCR and larval culture [31, 32, 50]. Additionally, the relatively long prepatent period as well as the dependence of the development of the infectious L3 on environmental conditions is responsible for the seasonality of *S. vulgaris* which may lead to false negative results at certain times of the year [23, 51]. Various studies were able to proof a seasonal fluctuation of egg shedding of *S. vulgaris*, with a peak in summer and a depression in winter [52–55]. Yet, the study at hand was not able to confirm such an influence of seasonality on the detection of *S. vulgaris*-eggs due to a non-seasonal collection of samples and a low number of positive samples. However, the present study revealed a significantly improved detection rate of *S. vulgaris* by real-time PCR in comparison to the current standard larval culture method.

A molecular method for the detection of *Strongylus* spp. was first described by Campbell et al., in 1995. The specificity of the conventional PCR investigating the ITS-2 gene of *S. vulgaris* has already been analysed by detecting interspecific variations in the sequence of the ITS-2 gene between *S. vulgaris*, *S. edentatus* and *S. equinus* via conventional PCR and subsequent sequencing [33]. The specificity of the real-time PCR was confirmed by Nielsen et al. [30] via cross reaction testing between *S. vulgaris*, *S. edentatus, S. equinus* and a mixture of different Cyathostominae larvae. Therefore, a possible cross reaction with DNA of other equine strongylid nematodes was not expected. Besides the specificity, the sensitivity of the real-time PCR has also been analysed by Nielsen et al. resulting in a detection limit of a 0.5 strongyle egg-equivalent [30].

Due to the developmental period of up to 14 days for strongyle larvae, the larval culture is a time-consuming [29] though cost-efficient method for the detection of *S. vulgaris*. A sensitivity of 73% and a specificity of 84% determined by larval culture compared to necropsy data have been reported by Nielsen et al. [28]. However, this method is also dependent on the presence of *S. vulgaris* eggs in the investigated samples just like the real-time PCR. Furthermore, the larval culture has the additional disadvantage that false negative results might occur due to an inhibited development of L3 which might be caused by fluctuations of temperature, humidity, fungal growth and contamination with free-living nematodes. In the present study, the successful cultivation of Cyathostominae larvae was verified by the positive correlation between the “number of Cyathostominae larvae” and the “FEC” of the faecal sample. Cyathostominae larvae were successfully cultivated in 98% of the strongylid egg-positive samples. A possible explanation for an inhibited development of larvae might be an accidental partial freezing of samples during transportation which was reported for 12 FEC-positive samples which revealed only a limited number of larvae in the larval culture. The negative impact of low temperatures on the development of strongyle larvae has already been descripted by Ogbourne and Duncan [23], Hasslinger [56] and Enigk [51]. Furthermore, steps of the procedure following cultivation such as discharging of supernatant, purification, sedimentation and pipetting might also lead to a loss of larvae. The morphological differentiation of larvae via microscopic examination needs to be conducted by qualified and experienced personnel, since the detection of *S. vulgaris* larvae is difficult and time-consuming [28]. False negative results due to the latter aspects might easily occur especially since *S. vulgaris*-positive samples often comprise only a limited number of *S. vulgaris* larvae. The usage of an aliquot procedure with the design to investigate 100–200 larvae in order to save time might also contribute to false negative results.

With the intent to avoid this error, all larvae per 10 g/faeces were identified in the present study.

In order to maintain or even reduce the low occurrence of *S. vulgaris* in Germany and to prevent the introduction of *S. vulgaris* within and among farms, an appropriate optimisation of the diagnostic and management procedures is crucial [3].

Application of real-time PCR for detection of *S. vulgaris* as a routine method is possible in any laboratory with appropriate equipment for the real-time PCR-procedure [45]. Strongyle eggs recovered by the standard combined sedimentation/flotation method can directly be used for the DNA-extraction and subsequent real-time PCR. In the present study, a FEC of 20 EPG was sufficient for the detection of *S. vulgaris* by real-time PCR.

Since the follow-up examinations in this study revealed that not every single examination reproduce an initially *S. vulgaris*-positive result, the investigation of composite faecal samples per horse by real-time PCR for an adequate detection of *S. vulgaris* might be useful in the daily routine diagnostics. Moreover, all horses of a herd or at least “high-risk patients” like newcomers and horses with unknown deworming history should be examined individually [3]. Preliminary to an integration into a new farm, thorough examination for *S. vulgaris* is also essential for horses originating from countries with a high prevalence for *S. vulgaris* like Denmark, a part of Italy and Poland [36, 43, 44].

## Conclusion

According to the results of the present study, the detection of patent infections with *S. vulgaris* via real-time PCR reveals a significantly improved detection rate improvement compared to the current standard method based on larval culture and subsequent morphological differentiation. Thus, the real-time PCR might be a reliable option for the detection of *S. vulgaris* in equine faecal samples in the routine diagnostic and in the integrated equine parasite control.

## Abbreviations

C_t_: Cycle threshold
DNA: Deoxyribonucleic acid
ELISA: Enzyme linked Immunosorbent assay
EPG: Egg per gram faeces
FEC: Faecal egg count
ITS: Internal transcribed spacer
L/10 g: Larvae per 10 g faeces
L1: Larvae-stage 1
L2: Larvae-stage 2
L3: Larvae-stage 3
PBS: Phosphate buffered saline
PCR: Polymerase chain reaction
SAT: Selective anthelmintic therapy
ZnSO_4_: Zinc sulphate solution
